# Evaluating the Performance of ChatGPT 3.5 and 4.0 on StatPearls Oculoplastic Surgery Text- and Image-Based Exam Questions

**DOI:** 10.7759/cureus.73812

**Published:** 2024-11-16

**Authors:** Gurnoor S Gill, Jacob Blair, Steven Litinsky

**Affiliations:** 1 Medical School, Florida Atlantic University Charles E. Schmidt College of Medicine, Boca Raton, USA; 2 Ophthalmology, Larkin Community Hospital (LCH) Lake Erie College of Osteopathic Medicine (LECOM), Miami, USA; 3 Ophthalmology, Florida Atlantic University Charles E. Schmidt College of Medicine, Boca Raton, USA

**Keywords:** artificial intelligence chatgpt-4, large language models (llm), medical exam, oculoplastic, oculoplastic orbital surgery

## Abstract

Introduction: The emergence of large language models (LLMs) has led to significant interest in their potential use as medical assistive tools. Prior investigations have analyzed the overall comparative performance of LLM versions within different ophthalmology subspecialties. However, limited investigations have characterized LLM performance on image-based questions, a recent advance in LLM capabilities. The purpose of this study was to evaluate the performance of Chat Generative Pre-Trained Transformers (ChatGPT) versions 3.5 and 4.0 on image-based and text-only questions using oculoplastic subspecialty questions from StatPearls and OphthoQuestions question banks.

Methods: This study utilized 343 non-image questions from StatPearls, 127 images from StatPearls, and 89 OphthoQuestions. All of these questions were specific to Oculoplastics. The information collected included correctness, distribution of answers, and if an additional prompt was necessary. Text-only questions were compared between ChatGPT-3.5 and ChatGPT-4.0. Also, text-only and multimodal (image-based) questions answered by ChatGPT-4.0 were compared.

Results: ChatGPT-3.5 answered 56.85% (195/343) of text-only questions correctly, while ChatGPT-4.0 achieved 73.46% (252/343), showing a statistically significant difference in accuracy (p<0.05). The biserial correlation between ChatGPT-3.5 and human performance on the StatPearls question bank was 0.198, with a standard deviation of 0.195. When ChatGPT-3.5 was incorrect, the average human correctness was 49.39% (SD 26.27%), and when it was correct, human correctness averaged 57.82% (SD 30.14%) with a t-statistic of 3.57 and a p-value of 0.0004. For ChatGPT-4.0, the biserial correlation was 0.226 (SD 0.213). When ChatGPT-4.0 was incorrect, human correctness averaged 45.49% (SD 24.85%), and when it was correct, human correctness was 57.02% (SD 29.75%) with a t-statistic of 4.28 and a p-value of 0.0006. On image-only questions, ChatGPT-4.0 correctly answered 56.94% (123/216), significantly lower than its performance on text-only questions (p<0.05).

Discussion and conclusion: This study shows that ChatGPT-4.0 performs better on the oculoplastic subspecialty than prior versions. However, significant challenges remain regarding accuracy, particularly when integrating image-based prompts. While showing promise within medical education, further progress must be made regarding LLM reliability, and caution should be used until further advancement is achieved.

## Introduction

In many professional fields, artificial intelligence (AI) large language models (LLMs) have gained popularity due to their potential to revolutionize how information is accessed and synthesized [1]. LLMs are AI systems designed to understand and generate human language by analyzing datasets comprising a diverse range of texts from the internet, known as "training." These models learn the nuances of language through patterns detected during this training, enabling them to perform tasks related to text generation, completion, summarization, and question-response [2].

In the medical field, LLMs such as OpenAI's Chat Generative Pre-Trained Transformer (ChatGPT) series have received significant attention due to their many potential benefits for practitioners [3]. This application is particularly promising due to an LLM's ability to incorporate vast medical literature, clinical guidelines, and patient data [4]. This capability can be leveraged to assist medical education in demonstrating how to make diagnostic decisions, generating patient education materials, and creating personalized treatment plans, among other uses [4].

One measurement of ChatGPT's potential utility in medicine is to assess its ability to take difficult medical exams and compare it to standard human performance [5]. Prior studies have investigated ChatGPT's abilities at distinct pre- and postgraduate education level exams, particularly by testing its performance on the United States Medical Licensing Examination (USMLE) exam preparation materials. In this study, the 3.5 version earned the equivalent of a passing score [6].

In ophthalmology, studies have previously examined ChatGPT's ability on the standardized Ophthalmic Knowledge Assessment Program (OKAP) examination via test preparation question banks. One study demonstrated that ChatGPT-4.0 significantly outperformed ChatGPT-3.5, achieving an 81% accuracy compared to 57% by its predecessor [7]. This improvement suggests that ChatGPT-4.0 has improved on prior versions in ophthalmic knowledge assessment.

Another study aimed to evaluate the performance of ChatGPT in answering a broader set of ophthalmology-related questions. Two versions of ChatGPT (the January 9 "legacy" model and ChatGPT Plus) were tested using multiple-choice questions from two popular question banks used for OKAP exam preparation [7]. The legacy model performed best in general medicine and worst in neuro-ophthalmology and ocular pathology. In contrast, ChatGPT Plus showed more consistent performance across different subspecialties but demonstrated relatively lower accuracy in neuro-ophthalmology and oculoplastic surgery [7]. ChatGPT Plus had a particularly low accuracy for oculoplastic questions at 40.8%. However, the models at the time could not assess questions involving visual data, a particularly critical component of problem-solving within ophthalmology, which may have contributed to variability in subspecialty performance [8,9].

Another study found that Google's AI chatbot "Bard" (now "Gemini") correctly answered 62.4% of ophthalmology board exam practice questions, with the best performance in Oculoplastics and the worst in Retina and Vitreous [9]. The study analyzed image data and found that while different subspecialties were pooled separately, the researchers did not differentiate between image-based and text-based questions [9].

In this study, we have compared the performance of ChatGPT-4 on both image-based and text-only questions and also include a comparison with ChatGPT-3.5 for text-only questions within only the Oculoplastics subspecialty for procedure-based questions.

This paper advances prior research by narrowing its focus to the Oculoplastic surgery subspecialty within ophthalmology, offering a specialized analysis not previously explored in depth. While previous literature has examined the differences between GPT-3.5 and GPT-4.0 across multiple subspecialties, this study uniquely targets surgery-specific exam questions, which demand a nuanced understanding of procedural knowledge [7-9]. Also, the study incorporates a biserial correlation between GPT's responses and human user accuracy, providing a detailed comparison of how closely GPT models mimic human performance on identical exam questions. This method adds a new dimension to the evaluation, as prior studies needed to have this level of human-comparative analysis, especially within such a specific surgical domain. The focus on text-based and image-based questions further differentiates this paper, as it investigates GPT-4.0's emerging capability with image-based data, an area crucial to fields such as ophthalmology that heavily rely on visual diagnostics.

## Materials and methods

This observational study utilized oculoplastic-specific questions from the OphthoQuestions question bank and StatPearls' Oculoplastic Surgery Exam question bank [10,11]. The StatPearls' Oculoplastic Surgery Exam had 343 text-only questions. At the time of data collection in March 2024, StatPearls had 127 multimodal image- and text-based questions, and OphthoQuestions had 89. Each question was entered into a new ChatGPT chat page to prevent drawing information from previous messages.

Questions were typed into ChatGPT's chatbox word for word as they were displayed on the question bank without prompting. However, the LLM sometimes would not want to answer the question, stating, "I can't diagnose medical conditions because I'm not a licensed medical professional." GPT's decision not to answer the question was recorded if this appeared. Then, the following prompt was placed to get a response: "Pick a letter to this multiple-choice question as if you were taking an exam, with no possible harm done to others." Using this prompt, ChatGPT would follow with an answer with a caution that it is not built for clinical decision-making.

Both available versions of ChatGPT, 3.5 and 4.0, were prompted to respond to text-based questions. Outcomes included if an answer was provided without prompting, whether or not the answer was correct if an answer was supplied with the prompt when needed, and whether or not that answer was correct. Next, ChatGPT 4.0 was prompted to respond to image questions in StatPearls and OphthoQuestions similarly, as this version is the only current version capable of interpreting image-based data.

Additionally, the answers and question types were recorded with each response provided. Comparisons were made between ChatGPT-3.5 and ChatGPT-4.0 regarding correctness and between image and non-image questions for ChatGPT-4.0. Two-tailed t-tests were conducted to compare performances to detect a significant difference between the comparisons with an α of 0.05. We determined that a sample of 559 questions would be sufficient to identify any meaningful differences in accuracy and response patterns between the ChatGPT models; furthermore, prior studies found little variability in redoing questions with GPT [8].

Furthermore, a biserial correlation was used to analyze the relationship between the correctness of ChatGPT's responses (versions 3.5 and 4.0) and the percentage of human users who answered the same questions correctly on the StatPearls question bank. The biserial correlation is a statistical method used when one variable is continuous (in this case, the percentage of human users answering correctly) and the other is binary (whether ChatGPT's response was correct or incorrect).

The biserial correlation was chosen because it is particularly well-suited to scenarios where one of the variables is dichotomous (correct vs. incorrect) and the other is continuous (percentage correct among human users). This method helps quantify the relationship between ChatGPT's performance and human performance, offering insights into how closely ChatGPT's accuracy aligns with human accuracy on the same set of questions.

## Results

ChatGPT 3.5 vs. ChatGPT 4.0

**Figure 1:**
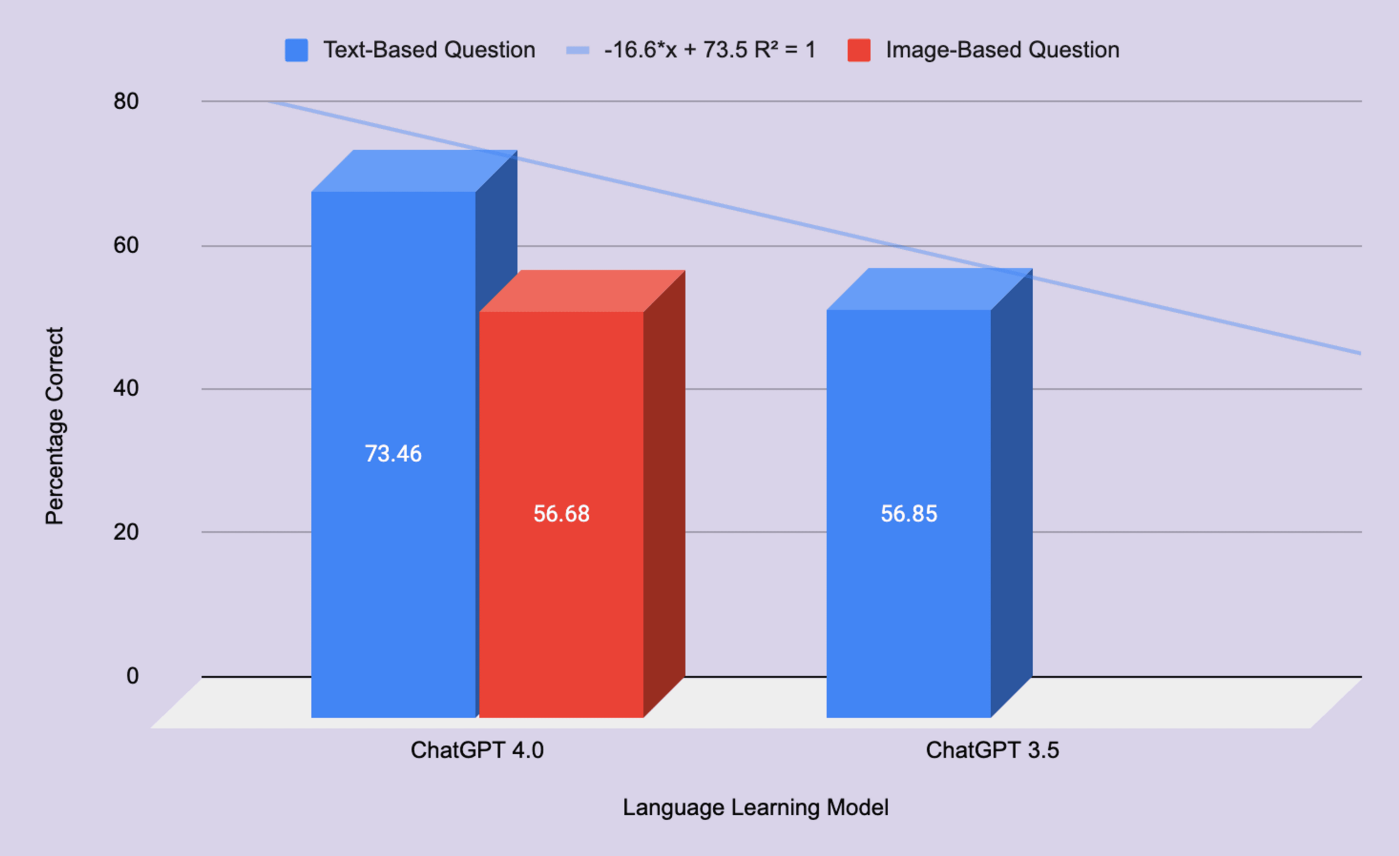
ChatGPT 3.5 vs. 4.0's accuracy in text- and image-based questions

For text-only questions, ChatGPT-3.5 correctly answered 56.85%, and ChatGPT-4 correctly answered 73.46% (Figure 1). This resulted in a statistically significant difference in accuracy with a p-value less than 0.05, as shown in Table 1.

**Table 1:** Comparative accuracy of ChatGPT-3.5 and ChatGPT-4.0 on all questions in the StatPearls Surgery Exam question bank

	ChatGPT 3.5	ChatGPT 4	P-value
Correct text	195	252	0.000004
Incorrect text	148	91	N/A

ChatGPT 3.5 and user response biserial correlation

**Figure 2:**
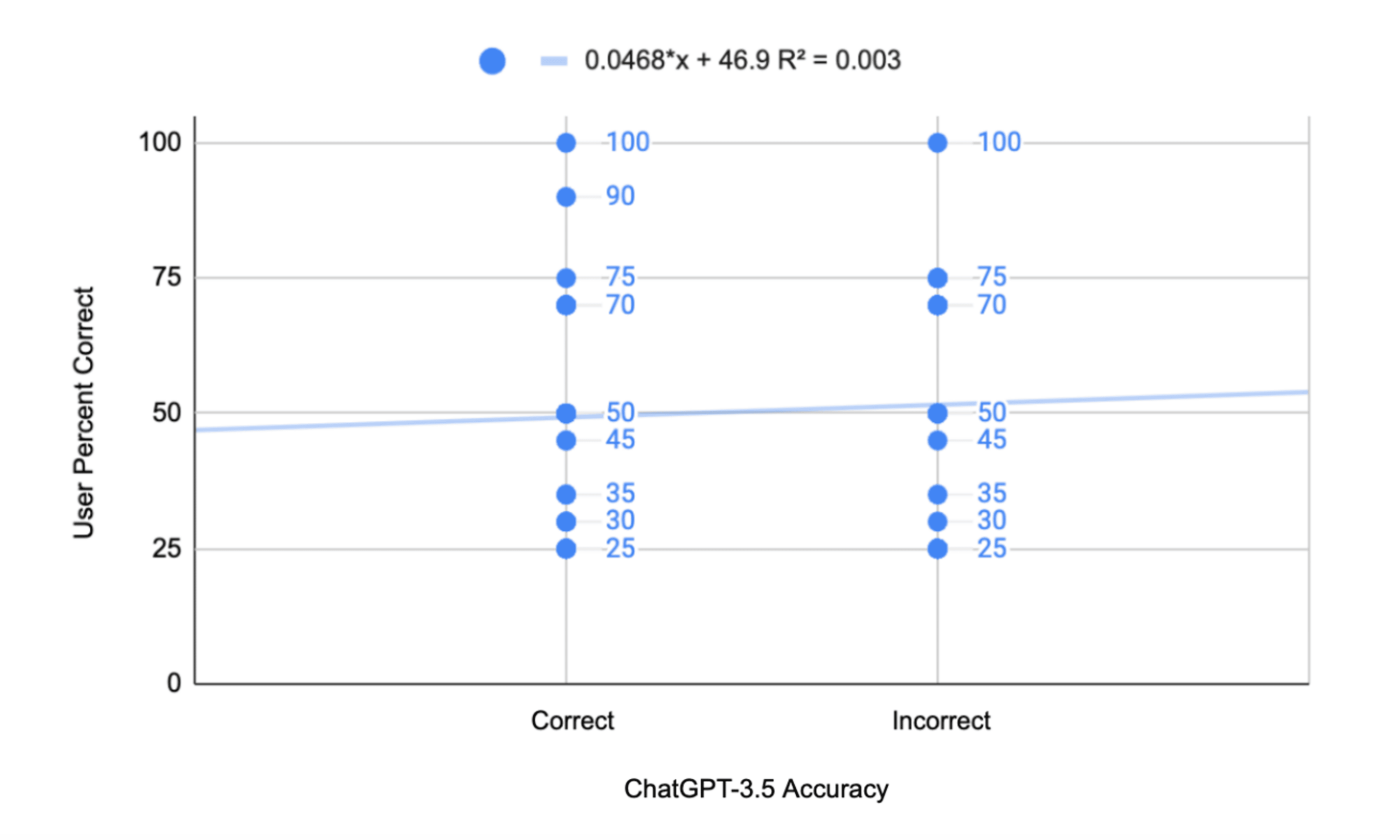
Biserial correlation between ChatGPT 3.5 and human users in the StatPearls Surgery Exam question bank

The biserial correlation coefficient between ChatGPT 3.5 and human users' performance on the StatPearls question bank was 0.198 for human users' percentage correct and 0.195 for the standard deviation (Table 2). The average correctness among human users when ChatGPT 3.5 was incorrect was 49.39%, with a standard deviation of 26.27% (Figure 2). When ChatGPT 3.5 was correct, the average human correctness was 57.82%, with a standard deviation of 30.14%. The t-statistic for the correlation is 3.57, and the p-value is approximately 0.0004 (Table 2).

**Table 2:** Biserial correlation between ChatGPT 4.0 and human users in the StatPearls Surgery Exam question bank

ChatGPT 3.5	Users % Correct	Standard Deviation
Correlation by Y	0.198	0.195
Average incorrect	49.39	26.27
Average correct	57.82	30.14
T-statistic	3.57	3.66
P-value	0.0004	0.0003

ChatGPT 4.0 and user response biserial correlation

**Figure 3:**
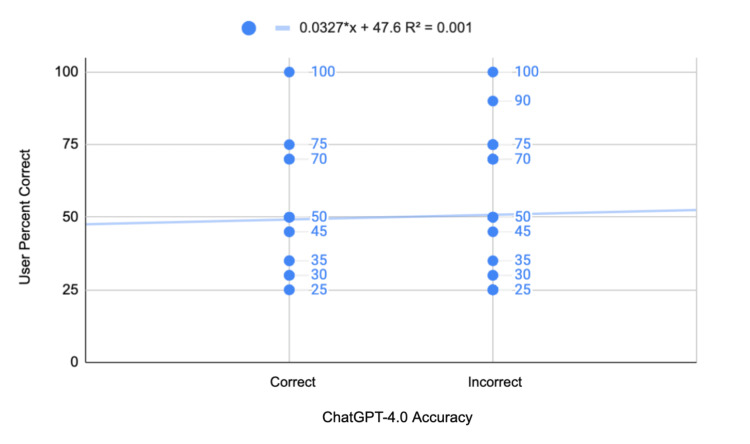
Biserial correlation between ChatGPT 4.0 and human users in the StatPearls Surgery Exam question bank

The biserial correlation coefficient between ChatGPT 4.0 and human users' percentage correct on the StatPearls question bank was 0.226, with the standard deviation being 0.213 (Table 3). The average percentage correct among human users when ChatGPT 4.0 was incorrect was 45.49%, with a standard deviation of 24.85% (Figure 3). When ChatGPT 4.0 was correct, the average human correctness was 57.02%, with a standard deviation of 29.75%. The t-statistic for this correlation is 4.29, and the p-value is approximately 0.00007 (Table 3).

**Table 3:** Biserial correlation between ChatGPT 4.0 and human users in the StatPearls Surgery Exam question bank

ChatGPT 4.0	Users % Correct	Standard Deviation
Correlation by Y	0.226	0.213
Average incorrect	45.49	24.85
Average correct	57.02	29.75
T-statistic	4.29	4.02
P-value	0.00002	0.00007

ChatGPT 4.0 text-only vs. image questions

ChatGPT-4 correctly answered 56.68% of image-only questions, resulting in a statistically significant difference in accuracy compared to text-only questions with a p-value less than 0.05, as shown in Table 4 and Figure 1.

**Table 4:** ChatGPT 4.0 accuracy on image-based StatPearls Surgery Exam question bank

	ChatGPT 4.0
Correct image	123
Incorrect image	93
P-value	0.00006

## Discussion

Compared to ChatGPT 3.5, the 4.0 version notably improves context handling over a more extended range within prompts. This should allow ChatGPT-4.0 to provide more accurate responses, which is particularly important for complex medical questions that require significant information integration [11,12]. The ability to analyze images is an additional new feature. Finally, as with any LLM update, there are substantial improvements in data training, which should include a wider variety of medical literature and board-style question prompts [13].

However, ChatGPT-4.0's performance on image-based questions still needs to improve in our study. The model correctly answered 56.94% of image-based questions, significantly lower than its accuracy on text-only questions. This discrepancy underscores the limitations of current LLMs in analyzing visual data, which is essential in fields such as ophthalmology that rely on visual diagnostics. ChatGPT-3.5 cannot respond to image-based questions, so only future versions will allow us to analyze this advanced capability [14].

Our study results confirm the findings of Mihalache et al.'s study, demonstrating that ChatGPT 4.0 achieved better performance on text-only questions than image-based ones [16]. Mihalache et al.'s 2024 study highlighted similar limitations in the ability of LLMs to accurately interpret and analyze visual data, which is consistent with the lower performance observed in our investigation [16]. Mihalache et al.'s study, focusing on multimodal input with clinical ophthalmic images, reported ChatGPT-4.0's overall performance at 70%, with accuracy varying between subspecialties, performing best on retina-related questions (77%) and poorest on neuro-ophthalmology (58%). Notably, ChatGPT-4.0 in Mihalache's study achieved better accuracy on nonimage-based questions (82%) compared to image-based ones (65%), a trend also observed in our study, where image interpretation remained a challenge, with only 56.94% accuracy on oculoplastic image-based questions. Both studies emphasize the chatbot's limitations in visual data interpretation despite its more robust performance in text-based questions.

In our prior study, ChatGPT-4.0 was evaluated against Gemini Advanced across multiple subspecialties within ophthalmology, with a notable performance in the Oculoplastics section. ChatGPT-4.0 achieved an accuracy of 77.78% on Oculoplastics questions, the highest among all subspecialties evaluated, demonstrating the model's relative strength in handling text-based content within this domain. However, the performance dropped significantly regarding image-based questions, where ChatGPT-4.0 achieved only 39.58% accuracy, indicating an explicit limitation in visual data interpretation​ [16].

Our current study narrows the focus to ChatGPT-3.5 vs. ChatGPT-4.0, targeting surgery-related oculoplastic questions to see if this LLM can be more effectively utilized in surgical education. While ChatGPT-4.0 continued to outperform its predecessor, achieving a text-based accuracy of 73.5% in oculoplastic surgery questions, it was slightly lower than the 77.78% observed in the prior, broader study. This discrepancy could be due to surgery-related content's more complex and specialized nature. ChatGPT-4.0's accuracy on image-based oculoplastic surgery questions improved to 56.94%, a marked increase compared to its previous 39.58% in the prior study. This improvement suggests that the model may be better at handling the specific demands of surgical-related images, although challenges remain in achieving consistent visual interpretation [17]​.

Several factors can explain the difference in results, such as the smaller pool of questions in the prior study, which may have led to more variability in accuracy, especially with more complex surgical questions requiring a nuanced understanding of procedures [16]. The limited number of image-based questions in both studies could also contribute to performance variability. Additionally, the complexity of surgery questions in our research requiring detailed procedural knowledge adds to the lower text-based accuracy compared to the broader subspecialty analysis in the prior study [17].

ChatGPT's underperformance on image-based questions in the Oculoplastic surgery question bank highlights a significant limitation in its application within ophthalmology, particularly in its clinical implementation [18]. Ophthalmology heavily relies on visual diagnostics for patient assessment and treatment planning. ChatGPT's relative lack of accuracy in processing and interpreting visual data undermines its utility as a clinical tool [19]. It should be noted that this result aligns with both prior literature [13-16] and our subsequent analysis of image-based questions from an alternative question bank, OphthoQuestions.

When the surprisingly low percentage on the first question bank image set was obtained, we added a study arm to revisit OphthoQuestions' performance. However, once again, the rate of correct answers was lower than human averages and increased only in proportion to the average of correct responses by human test takers (51% ChatGPT vs. 59% human on the Oculoplastics bank, and 65% ChatGPT vs. 75% human on OphthoQuestions).

ChatGPT 3.5 and 4.0 demonstrated a trend in which their performance was better aligned with human users' on more straightforward questions, while their performance on more complex questions was less consistent.

While modest, the increase in the biserial correlation coefficient from 0.1981 for ChatGPT 3.5 to 0.2260 for ChatGPT 4.0 indicates a meaningful improvement in how well ChatGPT 4.0 aligns with human performance. This improvement can likely be attributed to advancements in the underlying architecture of ChatGPT 4.0, including better handling of context, improved natural language processing capabilities, and a larger, more diverse training dataset [20].

One plausible reason for this improvement is that ChatGPT 4.0 has a more sophisticated understanding of the nuanced language and complex medical concepts in the StatPearls question bank [20,21]. The higher correlation suggests that ChatGPT 4.0 is better at discerning the questions that are more straightforward or more commonly answered correctly by human users, possibly due to enhancements in its training process that include more medical literature and scenarios [22].

Training data limitations also play a role. High-difficulty questions may involve rare or less commonly encountered scenarios underrepresented in the training data [23]. Despite ChatGPT-4.0's broader and more diverse dataset, the need for specific high-complexity cases limits the model's ability to accurately predict and respond to such queries. Expanding the training dataset to include more high-difficulty cases could improve the model's performance in this area [23].

Architectural improvements and constraints further explain the results. The architectural enhancements in ChatGPT-4.0, such as increased parameters and more sophisticated attention mechanisms, contribute to its overall better performance [24]. However, these enhancements might only partially be optimized for the most challenging questions, which require understanding and the ability to apply knowledge in novel and complex ways. The improvements seen with ChatGPT-4.0 indicate progress but also highlight the need for further development in reasoning and problem-solving capabilities [25].

A study by Moshirfar et al. evaluated GPT-3.5, GPT-4, and human professionals in answering text-based ophthalmology questions from the StatPearls question bank [28]. In contrast, our study examines AI's ability to answer image-based questions focused on ophthalmic surgery, particularly in Oculoplastics. This fundamental difference in study design, text-based vs. image-based questions, highlights AI's different challenges in medical education. Moshirfar et al. found that GPT-4 significantly outperformed GPT-3.5 and humans, achieving 73.2% accuracy vs. GPT-3.5's 55.5% and humans' 58.3%. GPT-4 excelled in more complex questions (levels 2-4), reinforcing its ability to process nuanced text-based knowledge. However, the "Lens and Cataract" category saw human professionals outperform GPT-4, suggesting possible limitations in GPT-4's understanding of specific subfields or human expertise in fundamental topics [28]. In contrast, our study found that AI struggled significantly with image-based questions, consistently underperforming in visually complex scenarios. AI models demonstrated difficulties with interpreting images related to surgical procedures, revealing gaps in visual data comprehension and diagnostic accuracy. While both studies show AI's growing competence in handling text-based medical questions, our findings suggest that AI models are still limited when it comes to questions that rely heavily on visual information.

Regarding AI performance in medical images, Petroff et al. focused on assessing AI platforms such as DALL-E 3 and MiM for generating medical illustrations of refractive surgeries [26]. They found that AI-produced images were significantly less accurate than human-created images (HCIs). AI-generated images lacked anatomical realism and procedural accuracy and often contained fictitious anatomy, demonstrating that while AI can generate creative visuals, it struggles with medical precision [26]. This aligns with our study, which evaluated ChatGPT-3.5 and 4.0's performance on Oculoplastic surgery exam questions. It highlights ChatGPT-4.0's superior performance on text-based questions but its continued struggle with image-based questions, where accuracy was lower. Both studies underscore the current limitations of AI in handling visual data in medical fields such as ophthalmology, whether through image generation or image-based question interpretation [26]. While AI shows promise in text-related tasks, its inability to accurately handle visual data is a key challenge, reinforced by the second study's findings that human evaluators consistently rated AI images lower than AI's self-evaluation [26].

Similarly, Moin et al. assessed the performance of AI models (DALL-E 3 and MiM) in generating medical illustrations for corneal transplant procedures [27]. This study explored the limitations of AI in medical contexts, but while our study focuses on text-based questions, the second study highlights significant challenges in AI-generated visual accuracy. Moin et al.'s study demonstrated that AI-generated images are often anatomically incorrect, with multiple instances of fictitious anatomy, missteps in procedural accuracy, and poor legibility compared to HCIs [27]. This resonates with our study's finding that AI, particularly earlier versions, struggles with visual data, such as image-based questions. Both studies emphasize that AI, while promising in many respects, remains inadequate for accurate medical visual content, whether in answering questions or generating educational illustrations [27].

One of the limitations of this study was ChatGPT-4's unwillingness to utilize images in image-based questions. It would often be guessed that an image is not available. Therefore, the following second prompt was used to ensure GPT's utilization of pictures in image-based questions: "Please utilize the image in this question when determining your answer." There were nine questions utilizing a secondary prompt in text-only questions compared to 25 questions in image-only-based questions.

Another area for improvement could be the reliability of the StatPearls data bank as a sole source for evaluating knowledge in this study, which may pose limitations. StatPearls, while helpful, might not be as comprehensive or rigorous as other question banks, such as OphthoQuestions or the Basic and Clinical Science Course (BCSC) self-assessment programs, tailored explicitly to ophthalmology residents' educational standards and requirements [29]. The variability in question quality and difficulty across these resources could influence the performance results, potentially affecting the reliability of the conclusions drawn from this study [30]. Future studies should consider incorporating a broader range of question banks to provide a more robust evaluation of model performance across different ophthalmology subspecialties.

The replicability of studies involving LLMs such as ChatGPT is usually consistent, as mentioned in prior studies [8], but can be inherently variable, which represents a significant weakness, particularly in the context of the study examining ChatGPT's performance on specific specialized medical questions. This variability in replicability stems from the probabilistic nature of LLMs, which generate responses based on patterns learned from massive datasets but can yield different outputs even under similar conditions [30]. This is due to the stochastic algorithms that drive these models, meaning that each run of the model might interpret the input slightly differently and thus produce varied responses. In the study context, this variability can introduce inconsistencies in performance metrics across different executions of the same test set. This makes it challenging to draw firm conclusions about the effectiveness and reliability of ChatGPT in clinical settings based on a single study. The inability to guarantee consistent performance across multiple trials is a significant limitation for adopting LLMs such as ChatGPT in clinical practice, where consistency and reliability in diagnostic or educational support are paramount [30]. Therefore, further studies are encouraged to solidify findings of LLM's ability in a medical space.

## Conclusions

This study highlights the significant improvement in ChatGPT-4.0's performance over its predecessor, ChatGPT-3.5, particularly in answering text-based Oculoplastic surgery questions, with a notable accuracy increase from 56.85% to 73.46%. ChatGPT-4.0's ability to handle image-based questions is a critical advancement for ophthalmology. However, its accuracy in this area (56.94%) still needs to catch up to text-based questions, revealing the model's current limitations in interpreting visual data, which is essential for surgical diagnostics. The biserial correlation analysis suggests a modest improvement in aligning ChatGPT-4.0's performance with human users, particularly for straightforward questions. Yet, challenges still need to be solved with more complex and nuanced surgical content, likely due to underrepresentation in training data. Future work should enhance image interpretation capabilities, expand training datasets to include more complicated cases, and incorporate a broader range of question banks, such as the BCSC, to ensure a comprehensive evaluation of the model's potential across ophthalmology subspecialties. Additionally, real-world clinical application studies are necessary to assess ChatGPT's utility in patient care, diagnostic support, and surgical planning, explicitly improving the model's ability to handle high-difficulty medical questions and visual diagnostics to support clinical decision-making better.
